# HSV-1 0∆NLS vaccine elicits a robust B lymphocyte response and preserves vision without HSV-1 glycoprotein M or thymidine kinase recognition

**DOI:** 10.1038/s41598-022-20180-0

**Published:** 2022-09-23

**Authors:** Grzegorz B. Gmyrek, Amanda N. Berube, Virginie H. Sjoelund, Daniel J. J. Carr

**Affiliations:** 1 Departments of Ophthalmology, The University of Oklahoma Health Sciences Center (OUHSC), 608 Stanton L. Young Blvd, DMEI PA415, Oklahoma City, OK 73104 USA; 2 Laboratory for Molecular Biology and Cytometry Research, The University of Oklahoma Health Sciences Center, Oklahoma City, OK 73104 USA; 3 Microbiology and Immunology, The University of Oklahoma Health Sciences Center, Oklahoma City, OK 73104 USA

**Keywords:** Live attenuated vaccines, Experimental models of disease

## Abstract

Effective experimental prophylactic vaccines against viral pathogens such as herpes simplex virus type 1 (HSV-1) have been shown to protect the host through T and/or B lymphocyte-driven responses. Previously, we found a live-attenuated HSV-1 mutant, 0ΔNLS used as a prophylactic vaccine, provided significant protection against subsequent ocular HSV-1 challenge aligned with a robust neutralizing antibody response. Yet, how the virus mutant elicited the humoral immune response relative to parental virus was unknown. Herein, we present the characterization of B cell subsets in vaccinated mice at times after primary vaccination and following boost compared to the parental virus, termed GFP105. We found that 0∆NLS-vaccinated mice possessed more CD4^+^ follicular helper T (T_FH_) cells, germinal B cells and class-switched B cells within the first 7 days post-vaccination. Moreover, 0∆NLS vaccination resulted in an increase in plasmablasts and plasma cells expressing amino-acid transporter CD98 along with an elevated titer of HSV-1-specific antibody compared to GFP105-vaccinated animals. Furthermore, O∆NLS-vaccine-induced CD4^+^ (T_FH_) cells produced significantly more IL-21 compared to mice immunized with the parental HSV-1 strain. In contrast, there were no differences in the number of regulatory B cells comparing the two groups of immunized mice. In comparing sera recognition of HSV-1-encoded proteins, it was noted antiserum from GFP105-vaccinated mice immunoprecipitated HSV-1 thymidine kinase (TK) and glycoprotein M (gM) whereas sera from 0∆NLS-immunized mice did not even though both groups of vaccinated mice displayed similar neutralizing antibody titers to HSV-1 and were highly resistant to ocular HSV-1 challenge. Collectively, the results suggest (1) the live-attenuated HSV-1 mutant 0∆NLS elicits a robust B cell response that drives select B cell responses greater than the parental HSV-1 and (2) HSV-1 TK and gM are likely expendable components in efficacy of a humoral response to ocular HSV-1 infection.

## Introduction

Herpes simplex virus type 1 (HSV-1) is a highly transmissible pathogen that establishes a latent infection in which greater than 3.5 billion individuals under the age of 50 are infected worldwide many of which are asymptomatic or present with mild symptoms typically associated with oral mucocutaneous lesions^1^. More recently, HSV-1 has established itself as the leading cause of genital HSV infection in developed countries^2,3^ and may contribute to the development or severity of Alzheimer’s disease^4^. Whereas there are no licensed vaccines against HSV-1 to date, there are numerous experimental prophylactic vaccine candidates including live-attenuated, recombinant, subunit cocktail, and epitope-based vaccines^5–10^. Most, if not all, of these vaccines elicit some form of adaptive immune response with the generation of memory T and B lymphocytes. Moreover, the attenuated or recombinant virus vaccines are designed to reduce or eliminate the establishment of latency in comparison to their parental, wild type counterparts which, in turn, would reduce or eliminate the possibility of reactivation.

The importance of the humoral response is unquestionable in defenses against viral infections including measles, rubella, and chicken pox viruses as resistance often depends on antibody levels^11^. B cells are central to the humoral response differentiating into antibody-secreting plasma cells. Indeed, in patients taking rituximab, an anti-CD20 monoclonal antibody which depletes peripheral B-cells and causes subsequent hypogammaglobulinemia, numerous viral infections are frequently reported^12,13^. Experimental studies have reported B-cell-deficient mice are less efficient in controlling HSV-1 infection with a higher incidence of encephalomyelitis and an increase in the establishment of viral latency^14–16^. This outcome may be influenced by the role of antigen presentation by B cells. Specifically, in HSV-1-infected B cell deficient mice, the number of CD4^+^ T cells that differentiate into IFN-γ-expressing CD4^+^ effector T cells are diminished whereas there are no significant changes in CD8^+^ T cell or NK cell populations^17^. Thus, B lymphocytes are a critical cell population in antibody production and antigen presentation that along with T cell-driven antibody class switching and affinity maturation in the germinal centers^18,19^ are instrumental in viral surveillance.

Draining lymph nodes are critical to the development of anti-viral immunity including T-dependent B cell class switching to IgG^20^. During the innate immune response to viral pathogens within the lymph node, type I IFNs are often expressed and facilitate the development of a Th1 response^21,22^ and maintain lymphatic vessel expression for afferent delivery of antigen^23^. However, type I IFN can have detrimental outcomes to the B cell response as they can drive terminal B lymphocyte differentiation to short-lived plasma cells depleting the anti-viral B cell population through the combination of the local activation of myeloid cells and the expression of TNF-α^24^. Thus, a fine balance between the anti-viral innate defenses and B and T cell activation in response to virus infection must be maintained to optimize the adaptive immune response including the generation of memory T and B lymphocytes.

We have begun to characterize the host immune response to a live, attenuated HSV-1, termed HSV-1 0∆NLS, used as a prophylactic vaccine against ocular HSV-1 challenge. This mutant virus lacks the nuclear localization signal of immediate early gene of infected cell protein 0 (ICP0) such that it is highly sensitive to the effects of type I IFN but remains highly immunogenic *in vivo*^25^. The efficacy has been linked to the humoral immune response as well as T cells that contribute to B cell help and CD8^+^ effector T cell activation and function^5,26^. In the present study, the B lymphocyte profile in the draining lymph nodes of HSV-1 0∆NLS vaccinated mice was conducted following the initial vaccine and boost. To determine if differences existed in the B cell response in vaccinating mice with an attenuated virus versus a fully competent virus, we compared the B cell profile of the HSV-1 0∆NLS to the parental KOS counterpart, GFP105. Following the initial vaccination, there was a significant increase in the number of CD4^+^ T follicular helper (T_FH_) and B cell subsets residing in the spleen and draining (popliteal) lymph node of the 0∆NLS vaccinated mice compared to the GFP105-vaccinated group. However, there was no change in antibody neutralization titers to HSV-1 following the initial vaccination or boost comparing the two vaccinated groups of mice. Likewise, there was no difference in efficacy in terms of preservation of the visual axis and lack of corneal pathology. However, two HSV-1 encoded proteins were not recognized by sera from 0∆NLS vaccinated mice including glycoprotein M and thymidine kinase. Furthermore, there was a significant reduction in recognition of glycoprotein C by 0∆NLS sera compared to sera from the parental virus vaccinated group in terms of peptide hits. Consequently, while mice vaccinated with the 0∆NLS attenuated virus display equivalent resistance to subsequent ocular HSV-1 challenge compared with GFP105-vaccinated animals, there were noted differences in the humoral immune response such that we would conclude glycoprotein M and thymidine kinase are not necessary as antibody targets for prophylactic vaccine efficacy against ocular HSV-1 challenge.

## Results

### Primary Vaccination with HSV-1 0∆NLS increases the number of T_FH_ cells and enhanced production of IL-21 that correlates with an increase in germinal center B cell formation

**Figure 1:**
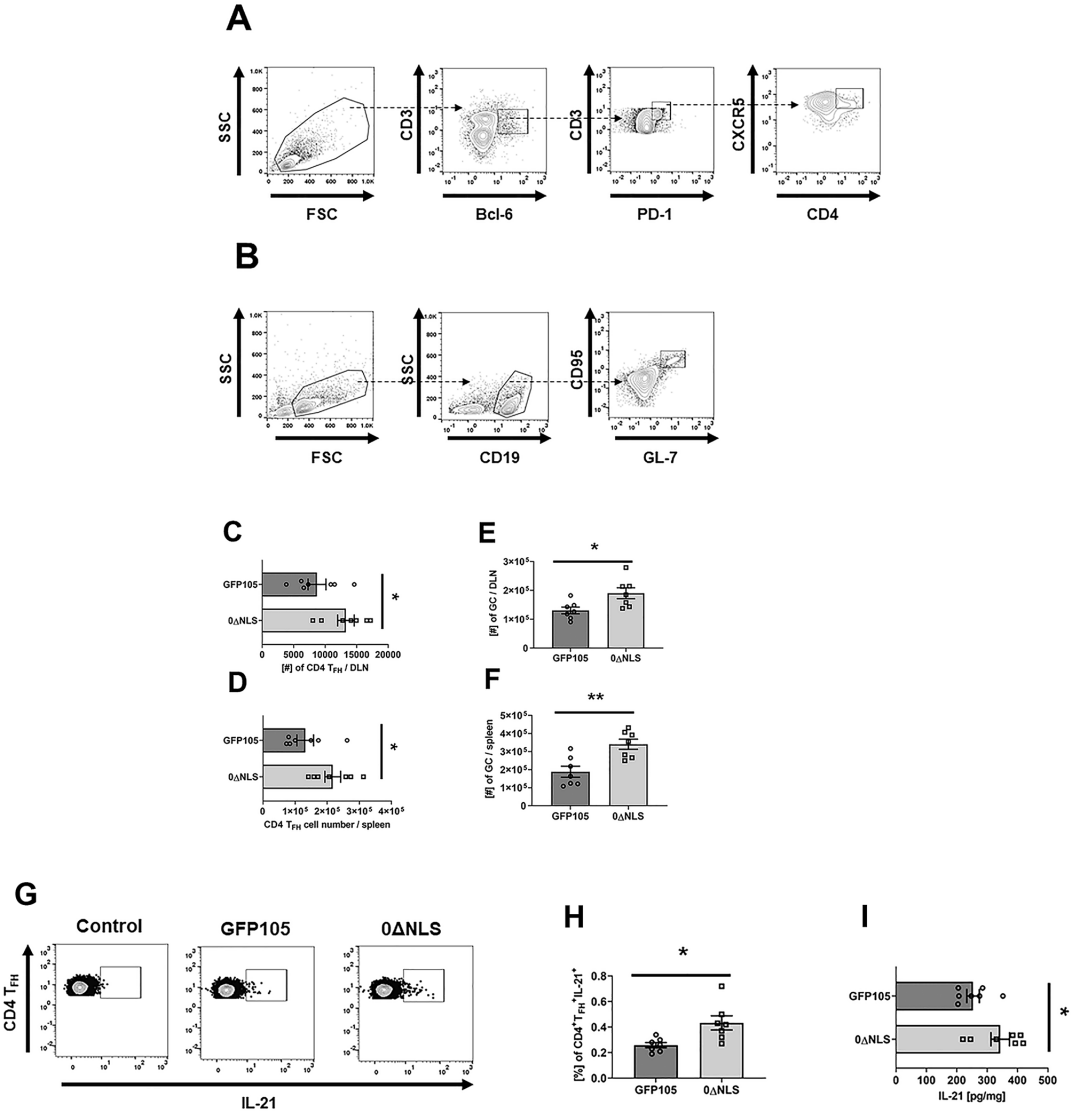
HSV-1 0∆NLS vaccination induces more CD4^+^ T_FH_ cells along with germinal center B cells. C57BL/6 mice (n = 6–7/group) were injected in the footpad with the HSV-1 parental (GFP105) or live-attenuated HSV-1 0ΔNLS at 10^5^ plaque forming units in 10 µl PBS. At day 7 or day 21 post immunization, the PLN and spleen were harvested and CD4^+^ T_FH_ cell and GC B cell numbers were assessed by flow cytometry. Panel A and B present gating strategy for discrimination of CD4^+^ T_FH_ cells and GC B cells, respectively. Panel C shows the CD4^+^ T_FH_ cell numbers found in the PLN at day 7 post-vaccination whereas panel D shows the CD4 + TFH cell numbers found in the spleen at day 21 post-vaccination. Panels E and F present the GC B cell numbers in the PLN at day 7 and in the spleen at day 21 post-vaccination, respectively. Panel G shows a representative flow cytometry profile of intracellular IL-21 cytokine staining in CD4^+^ T_FH_ cells after stimulation with PMA and ionomycin. Panel H summarizes the results showing frequency of CD4^+^ T_FH_ cells expressing IL-21. Panel I includes ELISA results quantifying the amount of IL-21 recovered in the PLN at day 7 post-vaccination normalized to tissue weight. The values are presented as mean ± SEM, **p < 0.01 and *p < .05 as determined by Student’s *t*-test.

T helper (T_H_) cells and dendritic cells play a critical role in the proliferation and differentiation of plasma cells and memory B cells initially taking place in distinct areas of the lymph nodes following tissue antigen (virus) exposure^27^. B cell proliferation within the follicles of the lymph node give rise to germinal centers, a specialized microanatomical region that provides an environment conducive to the selection of high-affinity antibody expressing B cells^18,28^. CD4^+^ T follicular helper (T_FH_) cells recruited to these sites through expression of CXCR5 facilitate germinal center (GC) B cell formation through interleukin (IL)-21 secretion^29,30^. To initiate our analysis of virus exposure to induction of B cell responses, we assessed whether vaccination of mice with HSV-1 0∆NLS resulted in changes in CD4^+^ T_FH_ cell defined as CD45^+^CD4^+^PD1^+^CXCR5^+^Bcl-6^+^ (Fig. 1A) and GC B cell defined as CD45^+^CD19^+^GL7^+^CD95^+^ (Fig. 1B) numbers compared to mice vaccinated with parental HSV-1 (GFP105) in the draining (popliteal) lymph node (PLN) at days 7 and 21 and spleen at day 21 post footpad administration. The results showed significantly more CD4^+^ T_FH_ cells and GC B cells in 0∆NLS vaccinated mice compared to those immunized with the parental HSV-1 GFP105 at the day 7 time point in the PLN, and day 21 post immunization in the spleen (Fig. 1C, D, and Fig. 1E, F). These results paralleled frequency findings as well. Specifically, a greater percentage of CD4^+^ T_FH_ and GC B cells were found in the PLN of 0∆NLS vaccinated mice at day 7 (13.09 ± 0.85% and 5.44 ± 0.71% respectively, *p < 0.05) compared to that in the PLN of GFP105 immunized animals (9.54 ± 1.02% and 3.46 ± 0.3%). Likewise, a greater percentage of CD4^+^ T_FH_ and GC B cells were found in the PLN of 0∆NLS vaccinated mice at day 21 (13.24 ± 1.01% and 2.71 ± 0.31% respectively, *p < 0.05) compared to that in the PLN of GFP105 immunized animals (9.56 ± 0.97% and 1.33 ± 0.23%). Consistent with these findings, the percentage of CD4^+^ T_FH_ cells expressing IL-21 from the PLN (Fig. 1G) was elevated in HSV-1 0∆NLS vaccinated mice compared to mice immunized with HSV-1 GFP105 at day 7 post immunization (Fig. 1H). This result equated to nearly twice as many IL-21-expressing cells from the PLN of HSV-1 0∆NLS vaccinated mice (58 ± 8) versus GFP105 immunized animals (31 ± 4). Likewise, IL-21 content in the PLN of HSV-1 0∆NLS vaccinated animals was significantly higher compared to HSV-1 GFP105 immunized mice (Fig. 1I). No differences were noted in the total CD4^+^ T cell count in the PLN or spleen comparing HSV-1 ∆NLS to GFP105 vaccinated animals (data not shown). Collectively, the results suggest HSV-1 ∆NLS promotes B cell expansion in the GC as a result of a specific increase in the number of IL-21 producing CD4^+^ T_FH_ cells in the PLN.

### Vaccination of mice with HSV-1 0ΔNLS results in an increase in the number of class-switched B cells associated with an increase in the titer of anti-HSV-1 antigen-specific IgG2b compared to HSV-1 GFP105 immunized animals

**Figure 2:**
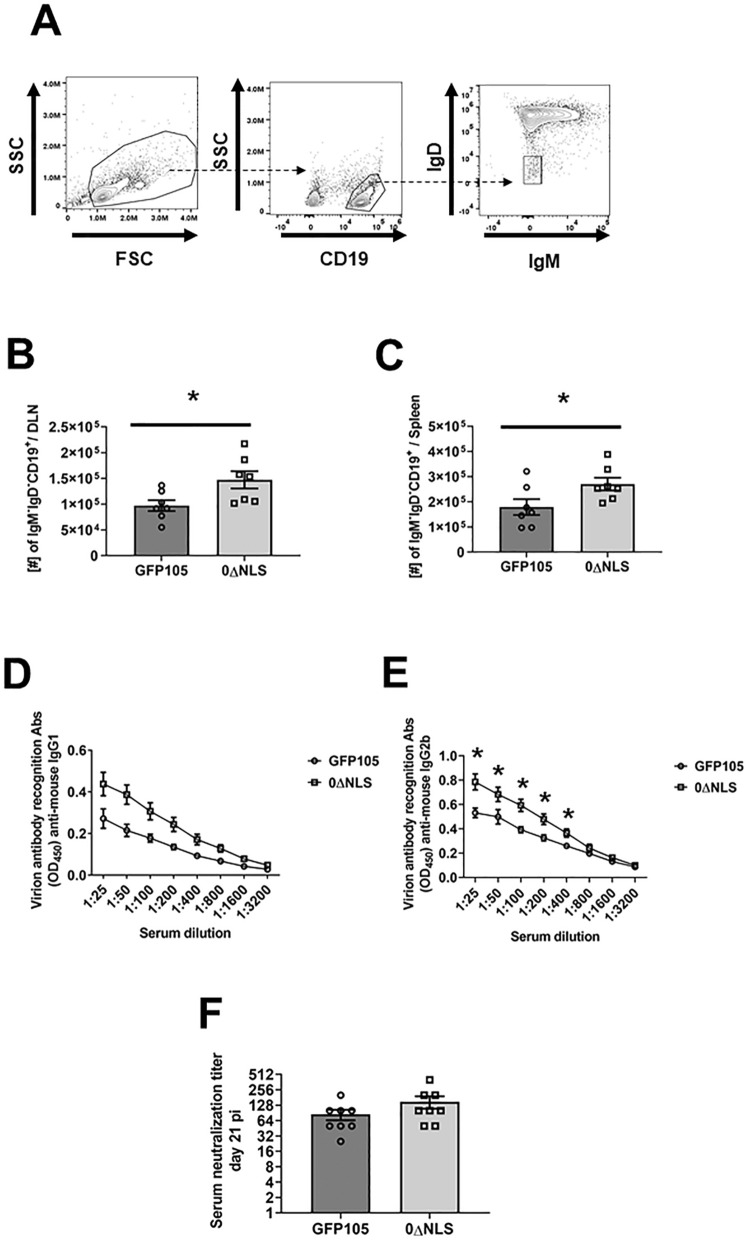
0∆NLS vaccination induces higher numbers of class-switch B cells along with an increase in HSV-1 IgG2b titers. C57BL/6 mice (n = 7–8/group) were injected in the footpad with the HSV-1 parental (GFP105) or live-attenuated HSV-1 0ΔNLS at 10^5^ plaque forming units in 10 µl PBS. Mice were euthanized at day 7 and day 21 post-vaccination and the number of class-switched B cells was determined at day 7 in the PLN and day 21 in the spleen post-immunization. At day 21 post-vaccination, sera were obtained and stored at –80 °C until it was assayed for anti-HSV-1 antigen reactivity and virus neutralization. Panel A presents a gating strategy for class-switched B cells (CD19^+^IgM^-^IgD^-^). Panels B and C present class-switched B cell numbers in the PLN and spleen of vaccinated animals, respectively. Panels D and E show anti-HSV-1 IgG1 and IgG2b immunoglobulin titers. Panel F presents antibody neutralization titers from sera of HSV-1 GFP105- and 0ΔNLS-vaccinated mice collected at day 21 post-immunization. The results are presented as the mean ± SEM, *p < .05 as determined by Student’s *t*-test.

Production of high affinity antigen-specific immunoglobulin as a result of somatic hypermutation and class switching often leads to efficient eradication of pathogens through neutralization and/or antibody-dependent cellular cytotoxicity. As a means to further study the dynamics of B cell activation following exposure to HSV-1, we next investigated class-switching defined by CD19^+^IgD^-^IgM^-^ B cells (Fig. 2A) in the PLN at day 7 or day 21 in the spleen post-vaccination. HSV-1 0∆NLS vaccinated mice were found to possess significantly more CD19^+^IgD^-^IgM^-^ B cells in the PLN and spleen at day 7 and day 21 post immunization, respectively (Fig. 2B, C). These results correlate with higher anti-HSV-1 IgG1 (Fig. 2D) and IgG2b (Fig. 2E) serum titers at day 21 post infection, although significance was noted only in case of anti-HSV-1 IgG2b (Fig. 2E). In contrast, there was no difference in the antibody neutralization titer to HSV-1 comparing mice vaccinated with 0∆NLS versus parental GFP105 (Fig. 2F). Thus, whereas 0∆NLS vaccinated mice display an elevation in class-switching that corresponds to increased anti-HSV-1 IgG2b titers, such results did not equate to an equivalent difference in antibody neutralization titers compared to GFP105 vaccinated mice suggesting a threshold in the level of anti-HSV-1 antibody titers has been reached in both vaccinated groups relative to virus neutralization. Nevertheless, the data does demonstrate an attenuated virus highly sensitive to type I IFN elicits a robust B cell antibody response that is equivalent to or greater than the non-attenuated parental virus when used as an immunogen to protect the host against ocular HSV-1 infection.

### HSV-1 0∆NLS vaccination induces higher number of plasmablasts and plasma cells expressing CD98

**Figure 3:**
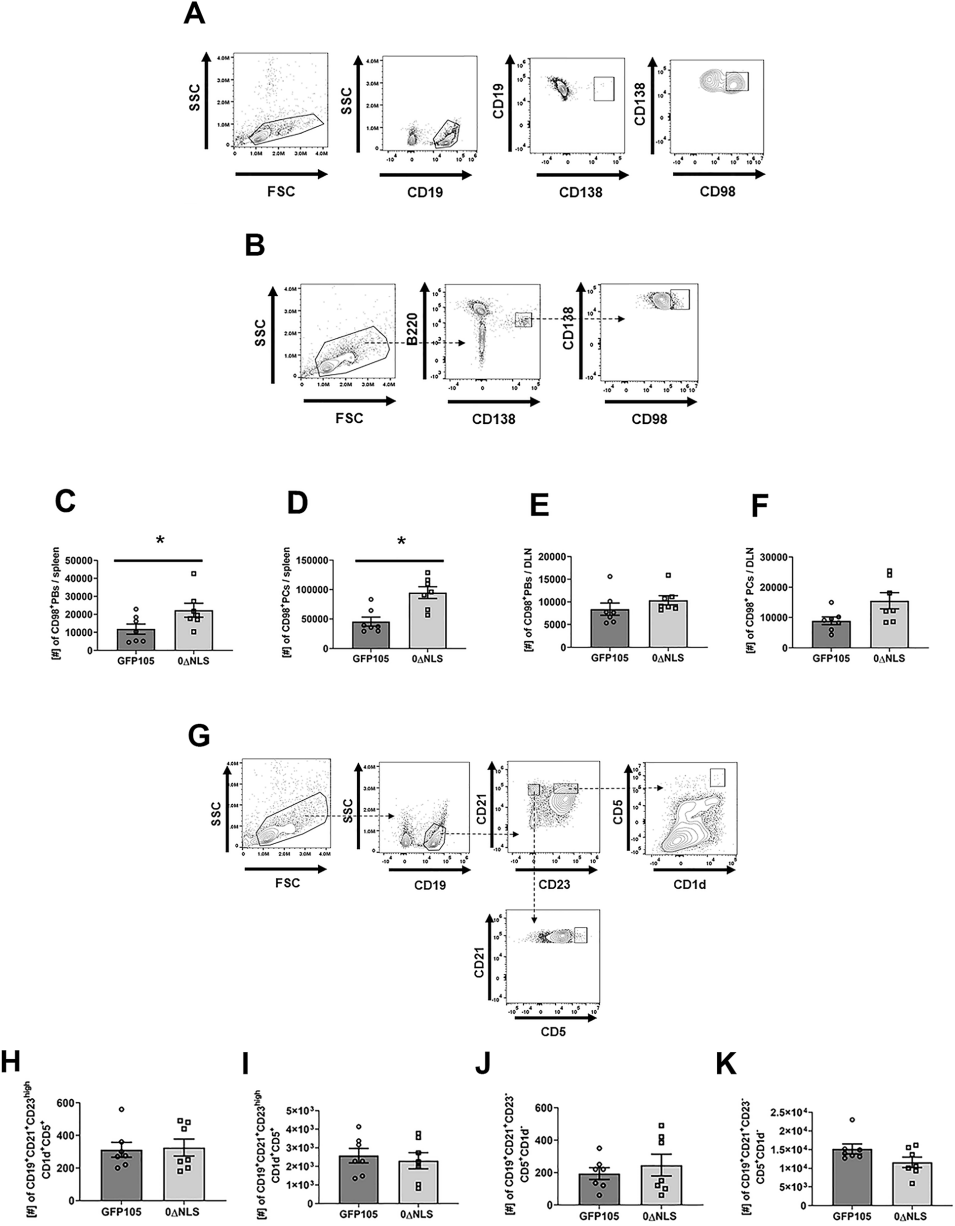
0∆NLS vaccination induces a greater number of plasmablasts and plasma cells expressing CD98 but no change in B regulatory cells in the spleen. C57BL/6 mice (n = 7/group) were injected in the footpad with the HSV-1 parental (GFP105) or live-attenuated HSV-1 0ΔNLS at 10^5^ plaque forming units (PFU) in 10 µl PBS. Mice were euthanized at day 7 and day 21 post-vaccination and the number of class-switched B cells was determined at day 7 in the PLN and day 21 in the spleen post-immunization. At day 7 and day 21 post-vaccination, the PLN and spleen were harvested and the number of plasmablasts (PBs) and plasma cells (PCs) expressing the CD98 marker were measured by flow cytometry. In addition, the number of CD19^+^CD21^+^CD23^high^CD5^+^CD1d^high^ (regulatory B cell, Breg) along with marginal zone Breg cells (CD19^+^CD21^+^CD23^-^CD5^+^CD1d^-^) were evaluated by flow cytometry as well. Panels A and B present a gating strategy for the evaluation of PBs expressing CD98 (CD19+CD138+CD98+) and PCs expressing CD98 (B220lowCD138+CD98+), respectively. Panels C and D show the number of PBs and PCs expressing CD98 in the spleen at day 21 post-vaccination respectively. Panel E and F show the number of PBs and PCs expressing CD98 in the PLN at day 7 post-vaccination, respectively. Panel G presents a gating strategy to discriminate Breg and marginal zone Breg cells. Panels H and I show the number of Breg cells in the popliteal lymph node at day 7 and in the spleen at day 21 post-vaccination, respectively. Panels J and K present the number of marginal zone Breg cells in the popliteal lymph node at day 7 and in the spleen at day 21 post-vaccination, respectively. The results are presented as the mean + SEM, *p < .05 as determined by Student’s t-test.

Antibody is produced by terminally differentiated B cells known as plasmablasts (PBs), which later give rise to plasma cells (PCs)^31^. The expression of the amino acid transporter CD98 has been shown to correlate with metabolic activity, longevity, antibody production, B cell proliferation and plasma cells formation^32–34^. In addition, the production of antibodies by long-lived plasma cells (LLPC) correlates with higher expression of CD98 as compared with short-lived plasma cells (SLPC)^34^. Given that the total number of PBs and PCs was not affected by vaccination (data not shown), we addressed how vaccination affect PBs and PCs in the context of their expression of CD98 at day 7 in the PLN and day 21 in the spleen post-vaccination. The expression of CD98 was found to be higher on PBs (CD19^+^CD138^+^, Fig. 3A) and PCs (B220^low^CD138^+^, Fig. 3B) in 0∆NLS-vaccinated mice resulting in significantly more of these cells in the spleen at day 21 post-vaccination (Fig. 3C, D respectively) but not in the lymph node at day 7 post-vaccination (Fig. 3E, F respectively). These results were also found to be true assessing frequency with a significantly greater percentage of PCs in the spleen of 0∆NLS-immunized mice at day 21 (35.29 ± 2.01%, *p < 0.05) compared to that found in the GFP105 immunized mice (19.66 ± 1.64%) but not that found in the PLN of 0∆NLS immunized mice at day 7 (6.19 ± 0.67%) compared to GFP105 vaccinated animals (4.66 ± 0.34%).

While most B cell subsets function are related to antibody production, cytokine secretion or antigen presentation, regulatory B cells (Breg) are known to possess immunosuppressive characteristics^35^. We characterized two Breg cell populations (Fig. 3G) that might have regulatory function including “classic” Breg cells (CD19+CD21+CD23+CD5+CD1d+) and a B cell population resembling marginal zone B cells (CD19+CD21+CD23-CD5+CD1d-)^36,37^. The data show both Breg cell subsets were modest in number in the draining lymph nodes at day 7 post-vaccination (Fig. 3H, I). However, by day 21 post-vaccination, both Breg cell populations were readily detectable in the spleen (Fig. 3J, K). Yet, at both time points evaluated there was no differences in the number of Breg cells residing in the PLN or spleen of GFP105 vs 0∆NLS-vaccinated animals suggesting that changes in PB and PC numbers were not reflected by Breg cell numbers in either the spleen or PLN of vaccinated mice. In summary, HSV-1 0∆NLS vaccine-induced animals develop a favorable B cell differentiation to PBs and PCs expressing CD98 over time evident by an increased number in the spleen. Such results are not apparent early on following vaccination in the PLN.

### Class-switched and germinal center B cell numbers and IFN-γ-secreting, HSV-1 gB-specific CD8^+^ T cell numbers remain elevated post-vaccination boost in the spleen of mice immunized with the 0∆NLS vs GFP105 vaccine

**Figure 4:**
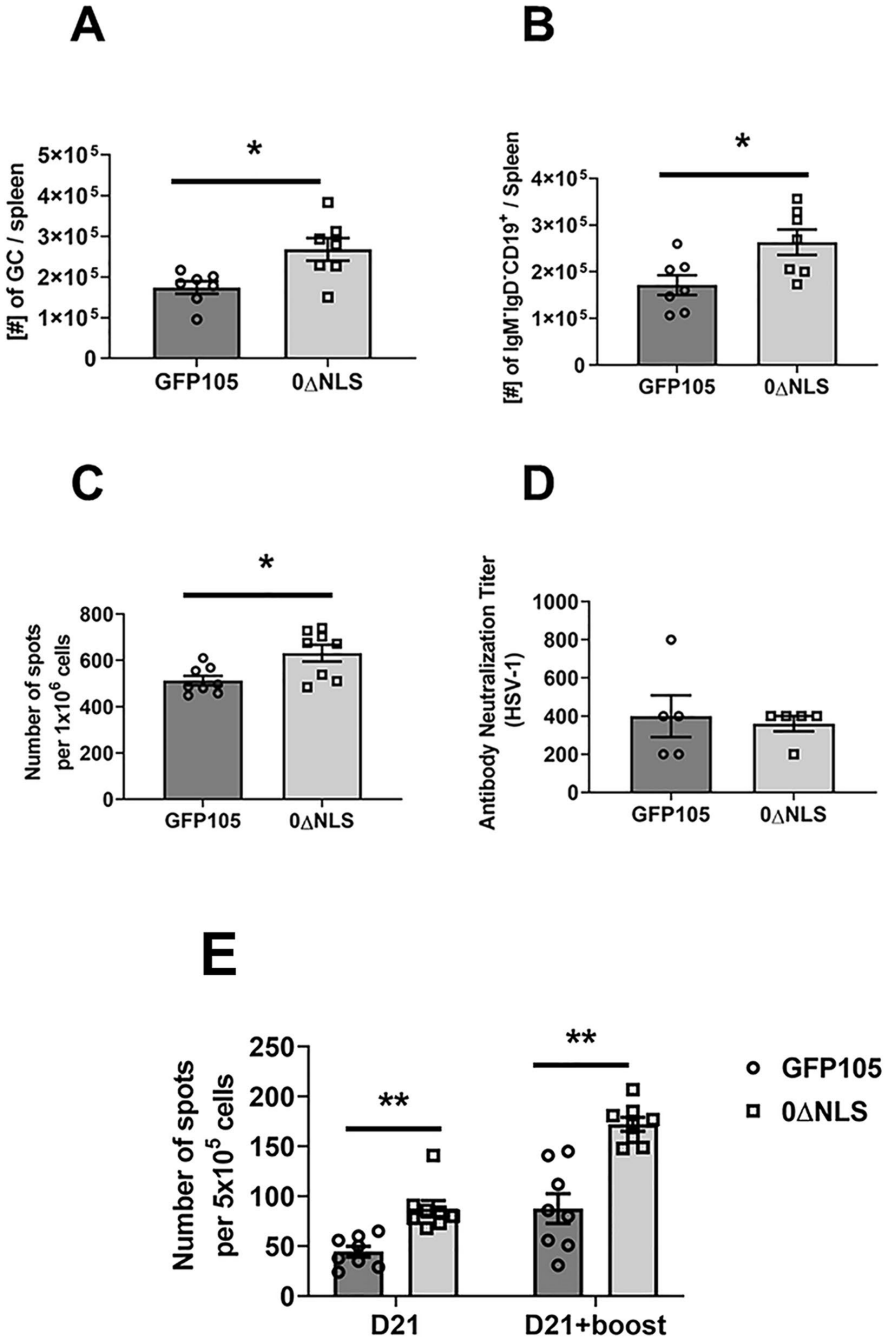
Primary immunization combined with boost vaccination results in an increase in the number of germinal center and isotype-switched B cells and IFN-γ-secreting, HSV-1 gB-specific CD8^+^ T cells in 0∆NLS vaccinated mice. C57BL/6 mice (n = 5–8/group) were injected in the footpad with the HSV-1 parental (GFP105) or live-attenuated HSV-1 0ΔNLS at 10^5^ plaque forming units (PFU) in 10 µl PBS. At day 21 post immunization, the mice were boosted with the same amount of either parental or live-attenuated HSV-1 virus and left for 30 days. Thirty days after boosting, mice were exsanguinated to evaluate (**A**) germinal center (GC) B cell (CD19^+^GL-7^+^CD95^+^) and (**B**) isotype-switched B cell (CD19^+^IgM^-^IgD^-^) formation along with (**C**) analysis of the number of pre-activated B cells secreting IgG1 after polyclonal stimulation, and (**D**) HSV-1 neutralization titer from sera obtained from mice 30 days post-booster. (**E**) The spleens were removed and processed to single-cell suspensions in which 1 × 10^6^ cells/well were stimulated overnight with gB peptide, SSIEFARL (10 µg/ml) to measure IFN-γ secreting CD8^+^ T cells by ELISPOT assay. Bars represent the mean ± SEM, **p < .01, *p < .05 comparing the indicated groups as determined by Student’s *t*-test.

Following primary vaccination, mice immunized with the 0∆NLS vaccine showed an increase in the number of GC B cells and class-switched B cells in the spleen compared to animals immunized with GFP105 21 days post-vaccination. To determine if this relationship changed following re-exposure to antigen in the form of a vaccine boost, mice vaccinated with 0∆NLS and GFP105 were boosted 21 days following the initial vaccination. Thirty days post-vaccine boost, the spleens of mice were evaluated for GC and class-switched B cell numbers. Consistent with the results following primary immunization, animals boosted with the 0∆NLS vaccine possessed significantly more GC (Fig. 4A) and class-switched (Fig. 4B) B cells as well as IgG1-secreting (Fig. 4C) B cells compared with animals boosted with GFP105. However, there was no difference in neutralizing antibody titers of sera obtained from GFP105- versus 0∆NLS-boosted animals (Fig. 4D).

Previously, we reported 0∆NLS-vaccinated mice displayed more functional HSV-1 gB-specific CD8^+^ T cells 7 days post-vaccination compared to mice immunized with GFP105^38^. Similar to the experimental design above, we wished to determine whether this relationship held following the booster immunization, mice vaccinated with GFP105 or 0∆NLS were evaluated for IFN-γ-secreting, HSV-1 gB-specific CD8^+^ T cells at 21 days post-primary immunization and 30 days post-boost. Similar to the results described for splenic B cells, there were nearly two times as many IFN-γ-secreting CD8^+^ T cells from the spleen of 0∆NLS-vaccinated mice compared to those immunized with GFP105 at the 21-day time point (Fig. 4E). Whereas the re-exposure to antigen in the form of a boost expanded this population in both vaccinated groups of mice 30-days post-boost, a significant increase in the number of IFN-γ-secreting, HSV-1 gB-specific CD8^+^ T cells from the 0∆NLS-vaccinated animals was noted (Fig. 4E). Therefore, the effect of 0∆NLS vaccination are not restricted to B cell populations but are found to influence functional CD8^+^ T cells as well.

### Visual axis remains intact in vaccinated mice following challenge with HSV-1

**Figure 5:**
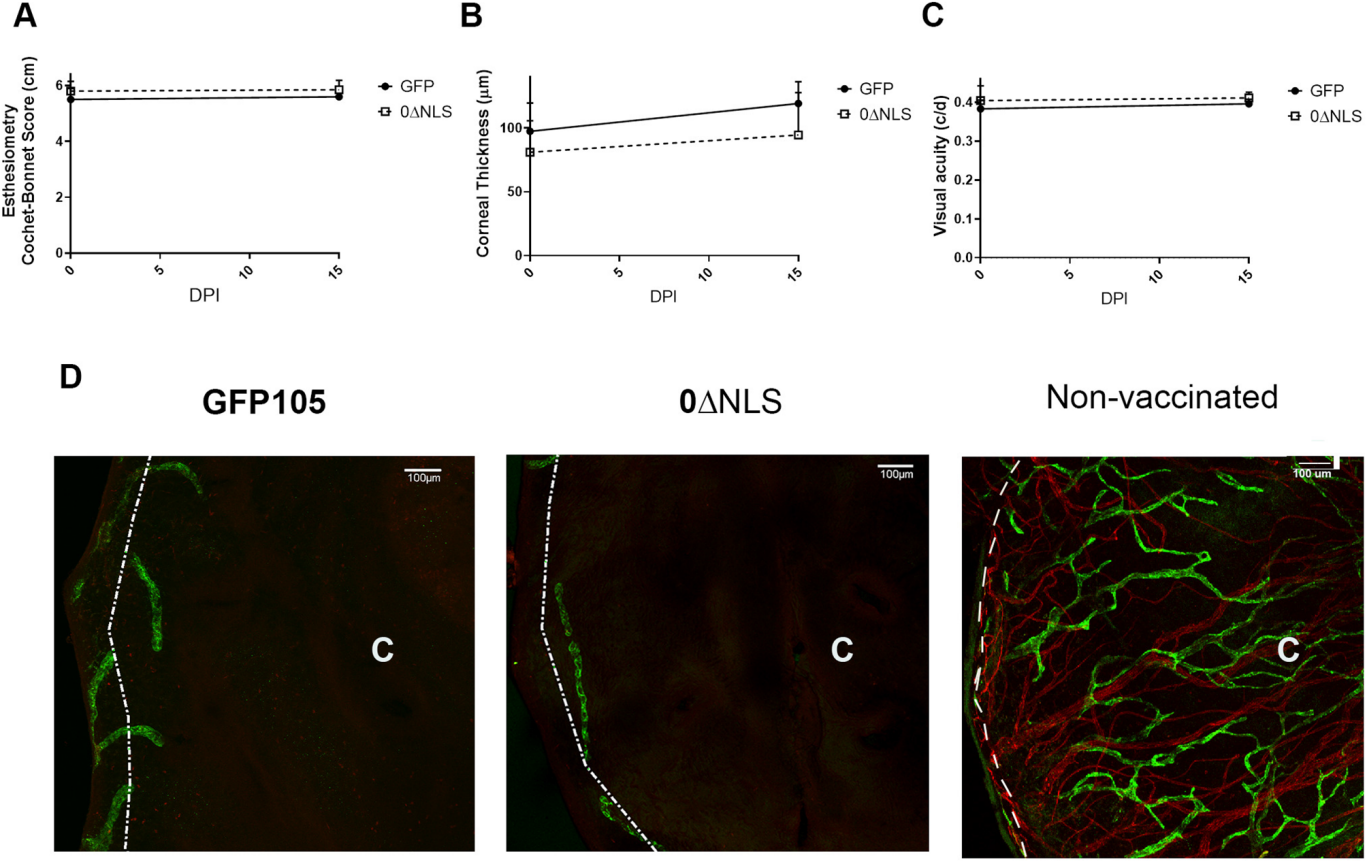
Visual axis is not compromised in vaccinated mice. C57BL/6 mice (n = 10/group) were injected in the footpad with the HSV-1 parental (GFP105) or live-attenuated HSV-1 0ΔNLS at 10^5^ plaque forming units (PFU) in 10 µl PBS. At day 21 post immunization, the mice were boosted with the same amount of either parental or live-attenuated HSV-1 virus and left for 30 days. Thirty days after boosting, mice were evaluated for mechanosensory function, edema, and peripheral vision (day 0) prior to challenge with HSV-1 (10^4^ PFU/cornea). At day 15 post infection, the mice were evaluated for (**A**) mechanosensory function measured by an esthesiometer, (**B**) cornea edema measured by optical coherence tomography and (**C**) visual acuity by optokinetic tracking. Mice were then exsanguinated and the corneas removed, processed, and stained for blood (red, CD31^+^) and lymphatic (green, Lyve-1^+^) vessels with images captured by confocal microscopy. A non-vaccinated mouse cornea stained in the same manner shows blood and lymphatic vessel genesis into the central cornea. The “C” indicates the location of the central cornea. The white dotted line depicts the limbus/cornea border. The scale bars are depicted at 100 µm.

In mice, acute ocular HSV-1 infection often results in cornea pathology including denervation^39,40^, opacity as a result of collagen fiber disruption^41^, inflammatory hypoxia^42^, local changes in substance P levels^43^, and products of infiltrating leukocytes^44^, hemangiogenesis^45^, lymphangiogenesis^46^, and edema^47^ leading to blindness or significant vision loss^48^. In previous work, we found a clear association with vaccine efficacy and preservation of the visual axis in terms of architecture, cornea integrity, and vision performance^26^. Specifically, HSV-1-elicited cornea denervation parallels the loss of mechanosensory function during acute infection^40^. Likewise, corneal thickness and reduction in vision acuity have been noted following HSV-1 cornea infection^26^. To determine whether there was any difference in efficacy of vaccinated mice to ocular HSV-1 challenge, GFP105- and 0∆NLS-vaccinated mice were infected with HSV-1 30 days post boost and evaluated for changes in the visual axis at 15 days post infection, a time when pathological changes within the cornea are evident and quantifiable^48^. We found no differences in the blink response associated with corneal denervation (Fig. 5A), edema (Fig. 5B), visual acuity (Fig. 5C), or neovascularization (Fig. 5D) comparing the vaccinated mice prior to and following infection. In the case of neovascularization (Fig. 5D), both GFP105- and 0∆NLS-vaccinated animals showed no corneal neovascularization in comparison to HSV-1-infected naive, non-vaccinated mouse underscoring the robust nature of protection in generating a protective immune response using the attenuated HSV-1 0∆NLS. Thus, GFP105- and 0∆NLS-vaccinated animals show no loss in visual function or pathology suggesting equivalent degrees of efficacy against ocular HSV-1 infection.

### Some HSV-1 encoded proteins are recognized by sera from GFP105- but not 0∆NLS-vaccinated mice

**Figure 6:**
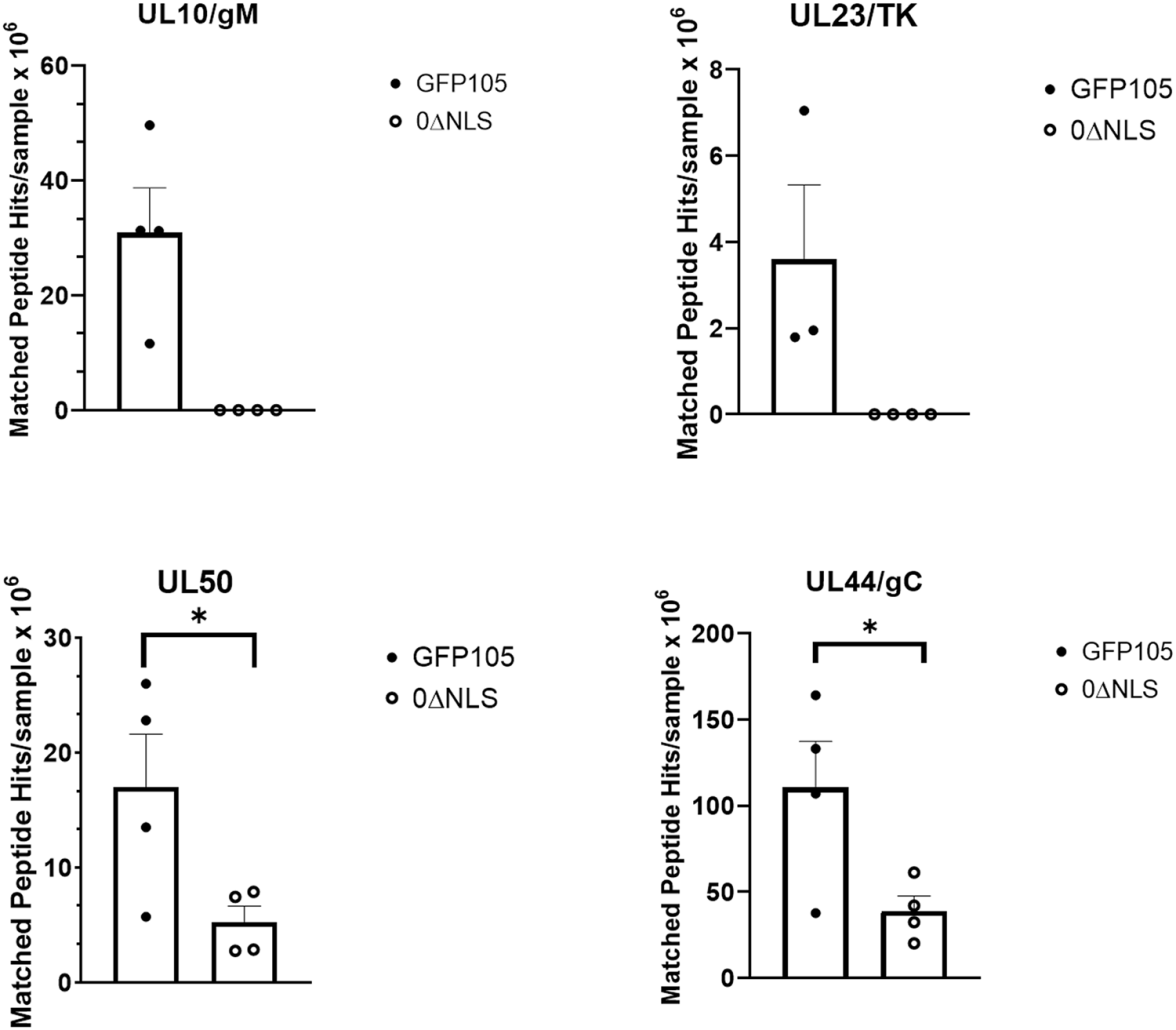
HSV-1 gM and TK are recognized by antiserum from GFP105- but not 0∆NLS-vaccinated mice. Serum (n = 4 samples/group) from GFP105- and 0∆NLS-vaccinated mice was used to immunoprecipitate virus-encoded proteins from HSV-1-infected Vero cell lysates. Precipitated proteins were analyzed by mass spectrometry. HSV-1 proteins were identified via cross-referencing derivative peptide ions with a reference sequence data base. The bars represent the mean matched peptide abundance/intensity per protein ± SEM, *p < .05 comparing the two groups as determined by multiple t-test using the Holm-Sidak method.

Previous studies by our group and others have reported on the importance of humoral (antibody) immunity against HSV infection^26,49^. Although we found no difference in the neutralizing antibody titer comparing GFP105- to 0∆NLS-vaccinated animals, there may be differences in the recognition pattern of antibodies to HSV-1-encoded proteins comparing the profiles generated from GFP105 and 0∆NLS sera immunoprecipitated samples. Therefore, we utilized mass spectrometry to identify virus-encoded proteins recognized by antiserum from GFP105- and 0∆NLS-vaccinated animals. The results show a total of 44 HSV-1-encoded proteins were identified from immunoprecipitated samples using GFP105 sera compared to 42 proteins from 0∆NLS sera. Two proteins uniquely precipitated by GFP105 sera included unique long (UL)10/glycoprotein (g)M and UL23/thymidine kinase (TK) (Fig. 6). Furthermore, two proteins immunoprecipitated by the GFP105 sera were found to have significantly higher peptide hits compared to that immunoprecipitated by 0∆NLS sera including UL50/dUTPase and UL44/gC (Fig. 6). No other HSV-1-encoded proteins identified by relative abundance of peptide hits were found to be different between GFP105 and 0∆NLS immunoprecipitated samples (data not shown). Of note, antibody recognition of proteins previously found to correlate with preservation of the visual axis including UL19/virion protein (VP)5, UL29/infected cell protein (ICP)8, UL27/gB, UL40/ribonuclease reductase subunit (RR)2, unique short (Us)6/gD, Us8/gE, UL1/gL, UL48/VP16, and UL12/alkaline nuclease (AN)^50^ were found to be recognized by antiserum from GFP105- and 0∆NLS-vaccinated mice suggesting these proteins may be critical in immune recognition to control acute ocular HSV-1 infection. We interpret the results to also suggest HSV-1-encoded TK and/or gM are not critical viral proteins to mount an antibody response to since the lack of recognition by antiserum from 0∆NLS-vaccinated mice did not lessen preservation of the visual axis.

## Discussion

There are many extrinsic and intrinsic factors that influence B cell survival, proliferation, differentiation, isotype switching and immunoglobulin secretion^51–55^. Cytokines are one set of factors that have a critical role in the regulation of B cell activation. For example, IFN-γ induces B cell proliferation as well as increases in IgG2 production^56,57^. Type I IFN augments B cell activation as well as differentiation to antibody-secreting cells and B cell receptor sensitivity^58,59^. Still, another cytokine IL-12, has been shown to promote differentiation of naive B cells into plasma cells^60^. In a previous study, we reported following initial vaccination with HSV-1 0∆NLS, an elevation in type I IFN and IL-12 production by cells was noted which drove an increase in IFN-γ-expressing CD8^+^ T cells in comparison to mice vaccinated with GFP105^38^. Thus, using the same vaccination model, changes in B cell subset formation may be linked to an altered cytokine microenvironment.

CD4^+^ T_FH_ cells play an important role in the regulation of T-cell dependent, B cell activation and differentiation. They are positioned within GC where they support the GC B cell activation, specifically B cell proliferation, class-switch recombination and differentiation of B cells into plasma cells^30,61,62^. In the present study, we report an increased number of CD4^+^ T_FH_ cells along with increased secretion of IL-21 by these cells. The importance of IL-21 in the regulation of B cell function has been described using IL-21 receptor-deficient mice. These mice were found to possess a compromised humoral immune response to T cell-dependent antigens. Specifically, GC B cells were found to be severely impaired resulting in an attenuated B cell proliferation response with a deficiency in plasma cell differentiation^63–66^. Similar to the current results, it was reported vaccination with another live-attenuated HSV-1 virus termed VC2 resulted in increased proliferation of CD4^+^ T_FH_ cells that correlated with robust antibody production and GC B cell activation^67^. Taken together, these studies underscore the strong influence of CD4^+^ T_FH_ production of IL-21 on GC B cell responses relative to PB and PC numbers as well as increased anti-HSV-1 antibody levels. While the mechanism that drives enhanced GC B cell activation is currently unknown, it is tempting to speculate it may involve levels of lymphotoxin generated by localized T cells as a previous study reported a loss in T_FH_ cells and impaired antibody response to HSV-1 using a lymphotoxin β receptor deficient mouse model^68^.

Our previous studies described changes in cell metabolism might be linked to effector function of CD8^+^ T cells. Relative to B lymphocytes, the activation and differentiation states of B cells may depend on their metabolism^69,70^. Long-lived plasma cells expressing amino-acid transporter CD98 utilize 90% of their cellular glucose not to generate ATP and metabolites but rather, for glycosylation of secreted antibodies^32^. The present study found mice vaccinated with 0ΔNLS possessed more PB and PC expressing CD98 in the spleen 3 weeks after primary vaccination in comparison to mice vaccinated with GFP105. Whether the 0∆NLS vaccination altered cell metabolism in the naive B cells driving proliferation and differentiation is currently unknown. CD98 deficiency has been reported to reduce B cell proliferation, PC formation, and antibody production; however, B cell proliferation does not depend on amino-acid transport function but rather CD98 supports integrin-dependent proliferation of B cells^34^. Further studies are needed to reveal whether an interconnected network of genes and metabolic pathways correlate with PC function after vaccination with HSV-1 0ΔNLS.

In addition to characterizing the initial B cell response following vaccination with 0∆NLS and GFP105, we also evaluated the response following vaccine boost 21 days post primary vaccination. Similar results in the B cell responses were noted. Furthermore, vaccinated mice retained and preserved their visual axis whether immunized with HSV-1 GFP105 or 0∆NLS. These results are critical for two reasons: (1) the attenuated HSV-1 0∆NLS elicits the same if not better humoral immune response as the fully virulent parental virus used as vaccines and (2) the immune response provides sufficient protection to retain the integrity and performance of the visual axis. Most experimental studies do not go into depth in terms of evaluating the cornea and how changes to its architecture may hinder the rodent’s peripheral vision. Denervation of the cornea in response to HSV-1 infection is a common response in mice^39,40^. Likewise, inflammation of the cornea as a result of HSV-1 infection in experimental animal models often leads to edema and neovascularization^23,71^. The culmination of these and other events can lead to a loss of peripheral vision as measured by optomotor kinetic tracking or OKT^26^. In the current study, the evaluation of these parameters in comparing HSV-1 0∆NLS to GFP105 demonstrates that the attenuated virus delivers the same degree of protection at a reduced risk of establishing latency compared to the parental virus^25^. Such results are consistent with equivalent neutralizing antibody titers to HSV-1 which we have previously reported is an essential component of a protective immune response associated with the HSV-1 0∆NLS vaccine^5^. However, upon evaluation of viral proteins recognized by antiserum from vaccinated animals, there were noted deficiencies in the recognition of some viral-encoded proteins in the 0∆NLS-vaccinated group. Specifically, 0∆NLS-induced antiserum did not immunoprecipitate HSV-1 gM or TK where sera from GFP105 immunized mice did. Moreover, recognition of HSV-1 UL50- and gC-encoded proteins based on peptide hits was reduced in the immunoprecipitated samples from 0∆NLS sera pulldowns. However, other virus-encoded proteins previously found to correlate with vaccine efficacy including VP5, ICP8, gB, RR2, gD, gE, gL, VP16, and AN^50^ were recognized by GFP105 and 0∆NLS antiserum. Thus, we conclude these proteins are important in the recognition by antibody to suppress virus replication and spread from the eye to the nervous system as well as prevent cornea pathology and preserve the function of the visual axis. In conclusion, the humoral immune response elicited by prophylactic vaccines is a critical component of providing resistance to subsequent HSV challenge reinforced in this study and others^49,72^. In the human patient, it has been noted HSV-1-specific memory B cells including class-switched memory B cells are correlated with asymptomatic HSV-1 seropositive individuals in comparison to symptomatic HSV-1 seropositive patients underscoring the likely contribution of humoral immunity in HSV-1 surveillance in the latent-infected human host^73^.

## Materials and methods

### Mice

Six-to-eight-week-old male and female C57BL/6 mice were obtained from Jackson Laboratory (Bar Harbor, ME) and housed under specific-pathogen free conditions in the animal vivarium at the Dean McGee Eye Institute. All procedures described under the methods section were approved by the University of Oklahoma Health Sciences Center animal care and use committee (protocol # 19–060 ACHIX). This study was carried out in compliance with the ARRIVE Essential 10 Guidelines.

### Mice infection/vaccination

Animals were vaccinated with parental GFP105 or live-attenuated 0ΔNLS HSV-1 derived from KOS strain^5^. Briefly, mice were footpad injected with 1 × 10^5^ PFU of GFP105 or HSV-1 0ΔNLS in 10ul of PBS. HSV-1 GFP105 has a GFP coding sequence on the immediate early ICP0 gene between codons 104 and 105 on exon 2 under a cytomegalovirus promoter, whereas HSV-1 0ΔNLS lacks the nuclear localization signal (NLS) motif, R-P-R-K-R-R, encoded by amino acids 501 to 508 on exon 3 on the GFP105 background. For tissue collection, mice were anesthetized by i.p. injection of ketamine and xylazine and blood collected prior to exsanguination. Alternatively, at day 21 post primary footpad immunization, anesthetized mice were immunized a second time in the ipsilateral flank (ID) with the same amount of virus described above. Thirty days post-booster, mice were anesthetized by i.p. injection of ketamine and xylazine and blood collected prior to exsanguination. The PLN and spleen were processed, and cells labeled as described below. Serum was fractionated using Microtainer serum separator tubes (Becton Dickinson, Franklin Lakes, NJ) and stored at −80 °C until used in serum immunoassays.

### IL-21 cytokine measurement

IL-21 cytokine content in tissue was performed with a commercially available pre-coated IL-21 ELISA kit plate (Thermo Fisher Scientific, Waltham, MA USA). PLN harvested at day 7 post infection were placed into 1.5 mL snap-capped Eppendorf tubes (Advanced Bullet Blender, Troy, NY) containing RIPA buffer (50 mM Tris–HCl, 1% NP-40, 150 mM NaCl, 1 mM EDTA, and protease and phosphatase inhibitors) and then homogenized using a Bullet Blender Storm 24 (Advanced Bullet Blender). The samples were then centrifuged (10,000 × g, 1 min), and cell-free lysates were collected and frozen at −80 °C until analyzed in the IL-21 ELISA assay. Intracellular cytokine staining within CD4^+^ T_FH_ cells was performed using a commercially available anti-mouse IL-21 antibody conjugated with PE (R&D Systems, Minneapolis, MN USA). Specifically, the lymph nodes collected at day 7 post-vaccination were passed through a 40 µm filter cell strainer (Midsci, St. Louis, MO) resulting in single-cell suspensions. Next, 3 × 10^6^ cells were stimulated with phorbol 12-myristate 13-acetate (PMA, 100 nM) and ionomycin (1ug/ml) for 6 h in the presence of brefeldin A (GolgiPlug, BD Biosciences). After the incubation period, cells were washed and stained with an antibody cocktail (containing anti-mouse CD45 conjugated with PerCP-Cy5.5, anti-mouse CD3 conjugated with PE-Cy7, anti-mouse CD4 conjugated with APC-Cy7, anti-mouse CXCR5 conjugated with BV421, and anti-mouse PD-1 conjugated with FITC) for 15 min at room temperature. Next, the cells were fixed, permeabilized, and stained with anti-mouse IL-21 conjugated with PE. The samples were then acquired with a MacsQuant flow cytometer (Miltenyi Biotec, Bergisch Gladbach, Germany) and analyzed with FlowJo software (BD Biosciences, Medford, OR).

### Isotype ELISA

IgG1 and IgG2b-specific HSV-1 antibody titers were measured by ELISA. Briefly, 96-well microtiter plates were coated with purified HSV-1 virions in carbonate buffer (pH 9.6) and incubated overnight at 37 °C. The plates were washed with PBS-polyoxyethylene (20) sorbitan monolaurate (Tween20, ThermoFisher Scientific, Waltham, MA) to remove excess antigen and serial dilutions of mouse sera were added to the wells followed by incubation at room temperature for 2 h. Next, the plates were washed with PBS-Tween and incubated with alkaline phosphatase-conjugated anti-mouse IgG1, or IgG2b detection antibodies (1:2000 dilution; Southern Biotechnology, Birmingham, AL) for 2 h at room temperature. Finally, the plates were washed with PBS-Tween and incubated with para-nitrophenyl phosphate substrate for 18 h at 37 °C. The optical density at 450 nm was measured using a Clariostar microplate reader (BMG Labtech, Ortenberg, Germany) with background correction at 540 nm.

### Antibody neutralization assay

Antibody neutralization assays were performed by exposing the confluent monolayers (50,000 cells/well of 96-well microtiter plate) of Vero cells (American Type Culture Collection, Manassas, VA) to 10,000 PFU HSV-1 (McKrae strain) in the presence or absence of serial dilutions of serum in the presence of guinea pig complement (Rockland Immunochemicals, Limerick, PA) for 2 h. Next, the serum dilutions were decanted, and Vero cells were incubated in RPMI 1640 medium supplemented with 10% heat-inactivated fetal bovine serum (FBS), 1 × antibiotic/antimycotic, and 10 μg/ml gentamicin (Gibco Life Technologies, Grand Island, NY) for 24 h. Neutralization titers were reported as the reciprocal serum dilution at which a 50% reduction in cytopathic effect is observed.

### Flow cytometry

CD4 T_FH_ cells and B cell subpopulation characterization was assessed by flow cytometry at day 7 and day 21 post primary footpad immunization or 30 days post-booster. Briefly, spleens were passed through a 40 µm filter cell strainer (Midsci) resulting in single-cell suspensions followed by treatment with multispecies red cell lysis buffer (Thermo Fisher Scientific, Waltham, MA) for red cell removal. Next, cells were washed and resuspended in PBS supplemented with 2% FBS (herein referred to as staining buffer or SB). PLN were harvested at the indicated time points and processed identical to spleens. One million spleen- or PLN-derived cells were resuspended in 100ul of SB and stained for the following cell population: follicular CD4^+^ T cells (CD45^+^CD3^+^CD4^+^PD-1^+^CXCR5^+^Bcl-6^+^), germinal B cells (CD45^+^CD19^+^GL-7^+^CD95^+^), plasma cells (CD45^+^B220^low^CD138^+^), plasmablasts (CD19^+^CD138^+^) class-switched B cells (CD45^+^CD19^+^IgM^-^IgD^-^), subsets and effectors of regulatory B cells (Bregs): marginal zone B cells (CD45^+^CD19^+^CD23^+^CD21^-^) and B10 (CD45^+^CD19^+^CD5^+^CD1d^high^). The samples with stained cells were acquired with MacsQuant flow cytometer (Miltenyi Biotec) or spectral flow cytometer Aurora (Cytek Biosciences, Fremont, CA) as described previously^38^. For assessment of HSV-1 gB-specific CD8^+^ T cell function, we utilized a procedure previously described by our group^74^. Acquired samples were exported as FCS files and further analyzed using FlowJo software version 10.8.1 (BD Biosciences, Medford, OR).

### ELISPOT assay for evaluation IFN-γ

ELISPOT for IFN-γ was described previously^38^. Briefly, 96-plate with immobilon-P membrane were coated overnight with primary IFN-γ Ab (BD Biosciences). Cells isolated from spleen of mice harvested at day 21 and at day 30 after boost, were plated at concentration 5 × 10^5^ cells per well followed by overnight re-stimulation with HSV-1 derived gB peptide, SSIEFARL (10 ug/ml). The plate was developed as described previously^38^.

### Visual axis assessment

Mechanosensory function, cornea edema and neovascularization, and visual acuity evaluations were performed by esthesiometry, optical coherence tomography, confocal microscopy, and optokinetic tracking responses as previously described^75^.

### Immunoprecipitation of HSV-1 proteins and identification by mass spectrometry

Sera obtained from GFP105- and 0∆NLS-vaccinated mice collected 30 days post-boost were used to immunoprecipitate viral proteins from HSV-1-infected Vero cell lysates as previously described^50^. Tryptic digestion of isolated proteins and mass spectrometry analysis was performed as previously described^50^.

### Statistical analysis

Statistical analysis was performed using GraphPad Prism (version 9.3.0). Data were shown as means ± standard error of the mean (SEM). Each figure legend describes the statistical test used for data analysis. Differences comparing GFP105- to 0∆NLS-vaccinated mice were considered significant with a *p* value < 0.05.

## Data Availability

The data sets generated and analyzed for this study are available from the corresponding author upon reasonable request.
